# Specific human endogenous retroviruses predict metastatic potential in uveal melanoma

**DOI:** 10.1172/jci.insight.147172

**Published:** 2022-05-09

**Authors:** Matthew L. Bendall, Jasmine H. Francis, Alexander N. Shoushtari, Douglas F. Nixon

**Affiliations:** 1 Division of Infectious Diseases, Department of Medicine, Weill Cornell Medicine, New York, New York, USA.; 2 Ophthalmic Oncology Service and; 3 Melanoma Service, Department of Medicine, Memorial Sloan Kettering Cancer Center, New York, New York, USA.; 4 Department of Medicine, Weill Cornell Medicine, New York, New York, USA.

**Keywords:** Microbiology, Oncology, Cancer

## Abstract

Uveal melanoma (UM) is a unique disease in that patients with primary UM are well stratified based on their risk of developing metastasis, yet there are limited effective treatments once metastases occur. There is an urgent need to better understand the distinct molecular pathogenesis of UM and the characteristics of patients at high risk for metastasis to identify neoantigenic targets that can be used in immunotherapy and to develop novel therapeutic strategies that may effectively target this lethal transition. An important and overlooked area of molecular pathogenesis and neoantigenic targets in UM comes from human endogenous retroviruses (HERVs). We investigated the HERV expression landscape in primary UM and found that tumors were stratified into 4 HERV-based subsets that provide clear delineation of risk outcome and support subtypes identified by other molecular indicators. Specific HERV loci are associated with the risk of uveal melanoma metastasis and may offer mechanistic insights into this process, including dysregulation of HERVs on chromosomes 3 and 8. A HERV signature composed of 17 loci was sufficient to classify tumors according to subtype with greater than 95% accuracy, including at least 1 intergenic HERV with coding potential (HERVE_Xp11.23) that could represent a potential HERV E target for immunotherapy.

## Introduction

Uveal melanoma (UM) is the most common primary intraocular cancer in adults (1, 2) with a stable incidence of 5.2 per million in the USA (3) and approximately 6 per million in Europe, with a gradient from Northern Europe, with 8–9 per million in Scandinavia, to Southern Europe, with less than 2 per million (4). UM arises from melanocytes along the uveal tract, mostly from the choroid but also from the iris or ciliary body (5). Risk factors for UM include a fair skin color, red or blond hair, light eye color, ocular melanocytosis and dysplastic nevi, cutaneous, iris or choroidal nevus, and familial syndromes, i.e., germline BRCA1-associated protein-1 (BAP1) mutations (1, 6). UM is more commonly seen in older age groups, with a progressively rising age-specific incidence rate that peaks at age 70 (1), but presents at a younger age in Chinese and Asian Indians than in Caucasians (7).

In contrast to cutaneous melanoma (CM), most UMs do not display any ultraviolet (UV) mutation signature and have a very low mutational burden (8), except for 2 subsets, 1 driven by germline MBD4 mutations, and 1 UV driven that is restricted to Iris UM (9). UM lacks BRAF, NRAS, and KIT alterations and instead harbors activating and mutually exclusive mutations in genes encoding the G-protein-alpha subunits GNAQ or GNA11 in 90% of primary tumors (10–12). Mutations in the CYSLTR2 or PLCB4 genes may be found in the remaining 10% of tumors (13, 14). These mutations lead to the activation of the MAPK and PI3K/AKT pathways (15) but also the transcriptional coactivator yes-associated protein (YAP) through both a Hippo-independent and Hippo-dependent circuit (3, 16, 17). In a few cases, secondary drivers might influence tumor development (18). Genomic profiling of UM metastases as opposed to primary tissue emphasizes the overrepresentation of BAP1 alterations (19). At the molecular level, 2–4 groupings have been identified by the analyses of gene expression (mRNA, ncRNA), DNA copy number, DNA-methylation, and somatic mutations (8).

The primary tumor is controlled by radiological and surgical interventions and local relapses are extremely rare. Nevertheless, approximately half of the patients develop metastases that rapidly progress to the fatal stage. Despite research, survival of patients with metastatic UM (mUM) has not changed over the decades. Thus, the major challenge in UM is the metastatic risk (20). The metastatic risk of each patient can be prognosticated, but the mechanisms of metastasis and pronounced liver tropism are still poorly understood.

Despite many attempts to treat mUM, survival of patients has not improved over 3 decades, although there are suggestions that survival for some patients can be extended. Therapeutic advances for mUM have lagged behind those for metastatic CM because mUM was thought to be unresponsive to immune therapy. Recently, however, IMCgp100 (tebentafusp), a manufactured T cell stimulator, shows promise in mUM and suggests that immune-based therapy can be effective (21–24). There has also been some success with tumor-infiltrating lymphocyte (TIL) therapy (25) and PD-1 inhibition (pembrolizumab) in MBD4-related hypermutator phenotype (26) or in combination with the histone deacetylase (HDAC) inhibitor entinostat in the pembrolizumab + entinostat (PEMDAC) phase 2 clinical trial (27). Single cell studies (28) suggest that there is a fairly rich infiltrating immune landscape in mUM and immune therapy, and targets for immune therapy are being investigated.

We and others believe that an important and overlooked source of novel neoantigenic targets comes from Human Endogenous Retroviruses (HERVs) (29–32). HERV expression is thought to be tightly controlled by intrinsic mechanisms, such as PIWI (33), APOBEC (34, 35), and other restricting mechanisms (36), which function to contain nucleic acids within a cell. Recent studies have shown that HERVs may be much more dynamically regulated than previously thought. In human cancers, HERVs have been suspected to play both pathogenic and beneficial roles (37–40). HERV expression has been reported in germ cell tumors (41–43), prostate (44–46) and breast (47–51) cancers, lymphoma (52, 53), and renal cell carcinoma (29, 54, 55) among others (56–61). HERVs have been studied for their ability to contribute to tumorigenesis by activating cellular oncogenes or mutating cellular tumor suppressor genes, resulting in aberrant gene expression that promotes a cascade of uncontrolled cellular proliferation (62, 63). HERV-encoded products (RNA, cDNA, and proteins), even with compromised functionalities, can be toxic, and their aberrant accumulation can contribute to various disease states, including cancer. However, there is substantial evidence that HERVs also contribute to cis-regulatory DNA elements and modify transcriptional networks for the good of the host, and they can also stimulate innate or adaptive responses (64–66). Thus, the study of HERVs in melanoma is at an early and crucial stage, and any potential contribution of HERVs to UM pathogenesis — and the corollary, of the potential for neoantigenic targeting — warrants further study.

We have been intrigued with recent studies that have identified specific HERVs as biomarkers for tumors responsive to treatment with checkpoint inhibitors (29), which leads to the question: could HERVs also be surrogate neoantigenic targets in metastatic UM, and if so, which HERVs? In particular, proviruses from the HERV-E family have been reported to express immunogenic antigens in renal cell cancer (54), thus prompting us to investigate locus-specific HERV-E expression in UM. Using our computer pipeline Telescope (67), we characterized the expression landscape of locus-specific HERVs within the Cancer Genome Atlas (TCGA) UM data set (*n* = 80). We identified specific differentially expressed (DE) HERVs and demonstrated that HERV expression predicted patient survival from metastases in a supervised machine learning model. The initial tumor microenvironment of UM is an immune sanctuary site within the eye, but interestingly, differential HERV expression before apparent metastatic spread can distinguish tumors which metastasize to those that do not, providing a new model for understanding metastatic spread.

## Results

### HERV expression landscape defines 4 UM subsets.

**Figure 1:**
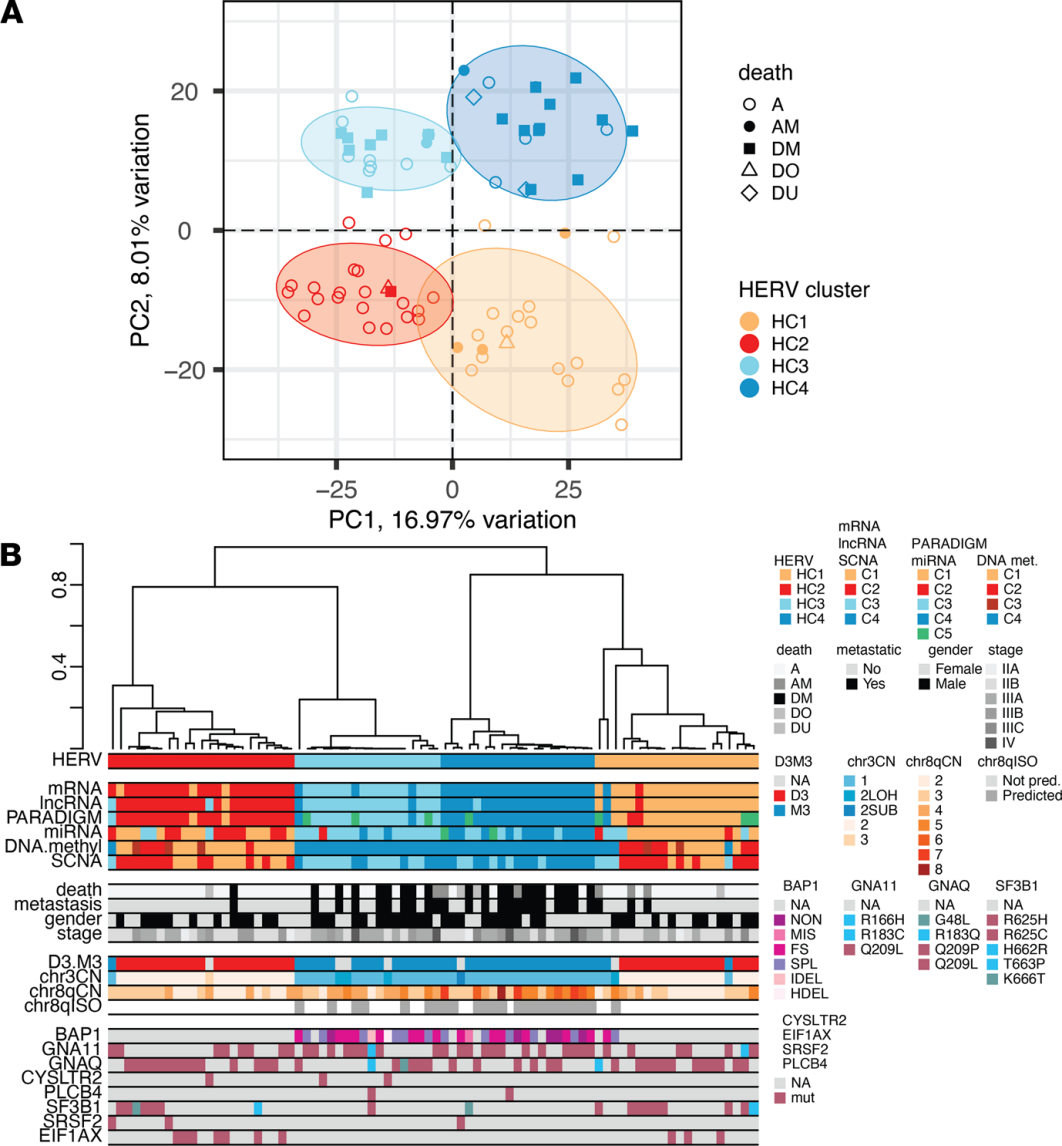
HERV-based clustering identifies 4 UM subsets. (**A**) Principal component analysis and unsupervised consensus clustering of HERV profiles. Shape indicates vital status or cause of death: A, alive; AM, alive with metastatic UM; DM, death caused by metastatic UM; DO, other cause of death; DU unknown cause of death. Statistical ellipses indicate a 90% confidence interval for each cluster. (**B**) Clustering dendrogram of HERV profiles with covariate tracks indicating (from top) alternate cluster assignments, clinical variables/outcomes, chromosome 3 and 8 copy number, and somatic mutations.

First, we sought to investigate whether locus-specific HERV expression signatures can be used for UM subtype classification and provide insights into molecular, clinical, and prognostic indicators. We used RNA-Seq data from 80 primary UM tumors from TCGA (phs000178) to profile expression of 14,968 HERV loci using our computer pipeline Telescope (67). Next, we performed unsupervised consensus clustering of HERV expression profiles to determine the number of UM subsets (parameter “k”) and membership of each sample. The consensus clustering approach identifies clusters that are robust to outliers by iteratively subsampling the data matrix and performing k-means clustering (for a given k) on the submatrix. The set of clusterings is then summarized by pairwise consensus, and the procedure is repeated for each value of k. The resulting cluster assignments are inspected using various visualization tools to select a reasonable cluster number and membership. Our analysis supports the identification of 4 or fewer UM subsets (k ≤ 4), while clustering solutions with k greater than 5 were less stable. In agreement with previous work on transcription-based classifiers (8), we selected a 4-cluster classification (Figure 1A). These 4 HERV subsets corresponded with 2 previously characterized prognostic classes. HERV clusters 1 and 2 (HC1 and HC2, hereafter referred to as HC1/2) correspond with low metastatic risk class 1 UM tumors while HC3 and HC4 correspond with poor prognosis and high metastatic risk class 2 UMs. Most UMs in HC1/2 have disomy 3 (D3) status (37/43) and do not have BAP1 alteration (41/43), while no D3 UMs are found in HC3/4 and only 4 out of 37 lack BAP1 mutations. Few HC1/2 primary UMs later developed metastases (3/20 and 1/22, respectively), while half of the HC3 clusters (9/18) and the majority of the HC4 clusters (13/19) developed metastases (Figure 1B).

Next, we asked whether clusters identified from HERV expression profiles are associated with other molecular classifiers or clinical outcomes. Using the adjusted Rand index (ARI) and Fisher’s exact test, we found that HERV clusters were most similar to clusters based on mRNA and lncRNA expression profiles (ARI = 0.747, *P* = 2.13 × 10^–34^ and ARI = 0.747, *P* = 5.49 × 10^–33^, respectively). HERV clusters were also significantly associated with clusters based on somatic copy number alterations (ARI = 0.300, *P* = 2.68 × 10^–16^), DNA methylation (ARI = 0.303, *P* = 2.09 × 10^–15^), miRNA (ARI = 0.321, *P* = 1.06 × 10^–15^), and PARADIGM pathway analysis (ARI = 0.739, *P* = 1.09 × 10^–29^). HERV-based classification was also significantly associated with important clinical and prognostic indicators, including D3/monosomy 3 (D3/M3) status (Fisher’s exact test, *P* = 2.649 × 10^–16^), metastasis (*P* = 7.076 × 10^–6^), and cause of death (*P* = 1.662 × 10^–7^), while not associated with gender (*P* = 0.7992).

**Figure 2:**
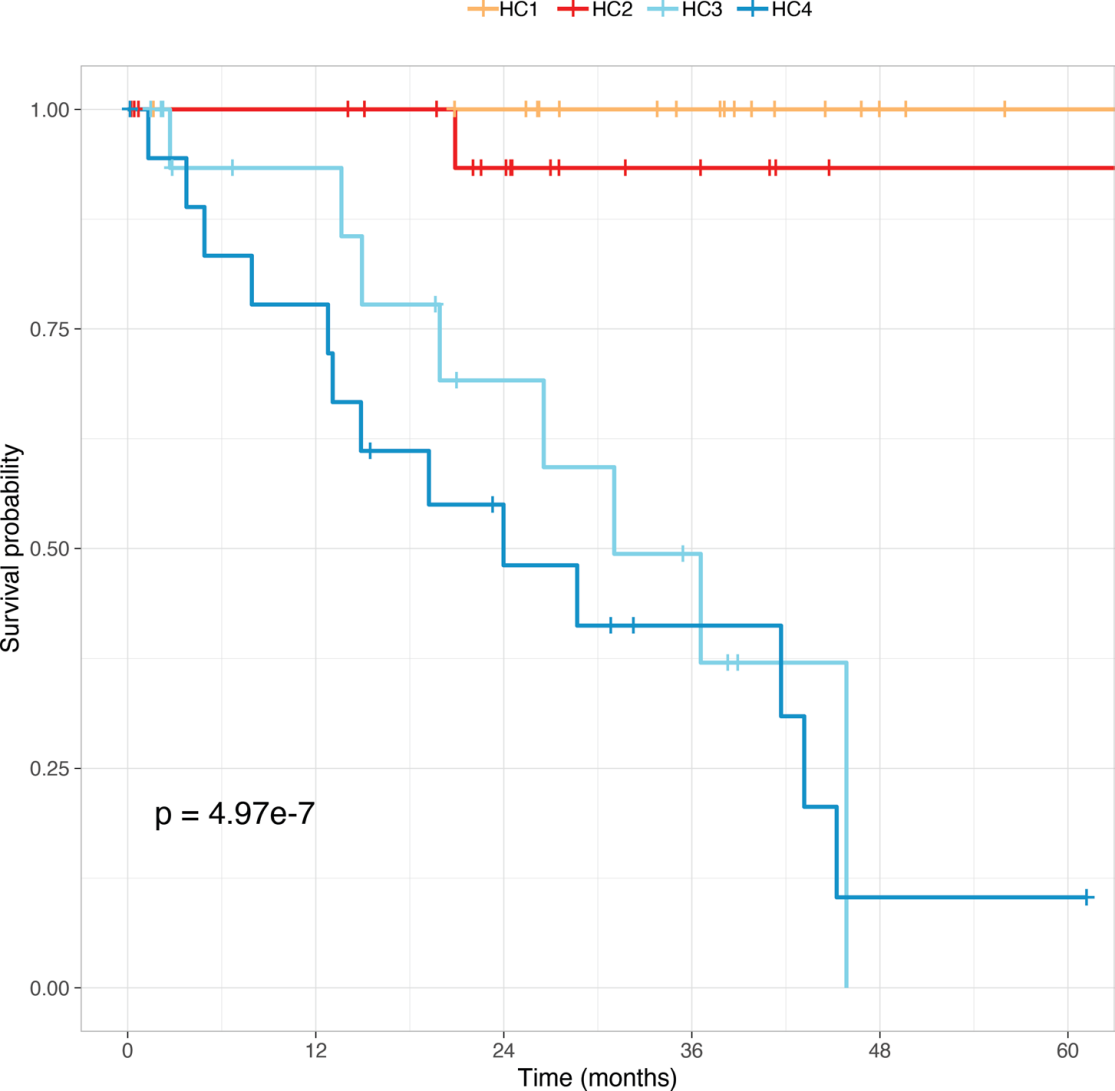
Kaplan-Meier survival analysis of HERV UM subtypes. Intervals to clinical outcome were calculated for documented death due to mUM or last follow-up, excluding other causes of death (*n* = 78).

We then examined the relationship between HERV cluster subtypes and metastatic death using Kaplan-Meier analysis (Figure 2). Intervals to clinical outcomes were calculated for documented death due to mUM or last follow-up, excluding other causes of death. The HERV subtype was significantly associated with clinical outcomes (*n* = 78, *P* = 4.97 × 10^–7^), with clusters HC3 and HC4 associated with a higher risk of death.

### Specific HERV loci are differentially expressed in UM subsets.

**Figure 3:**
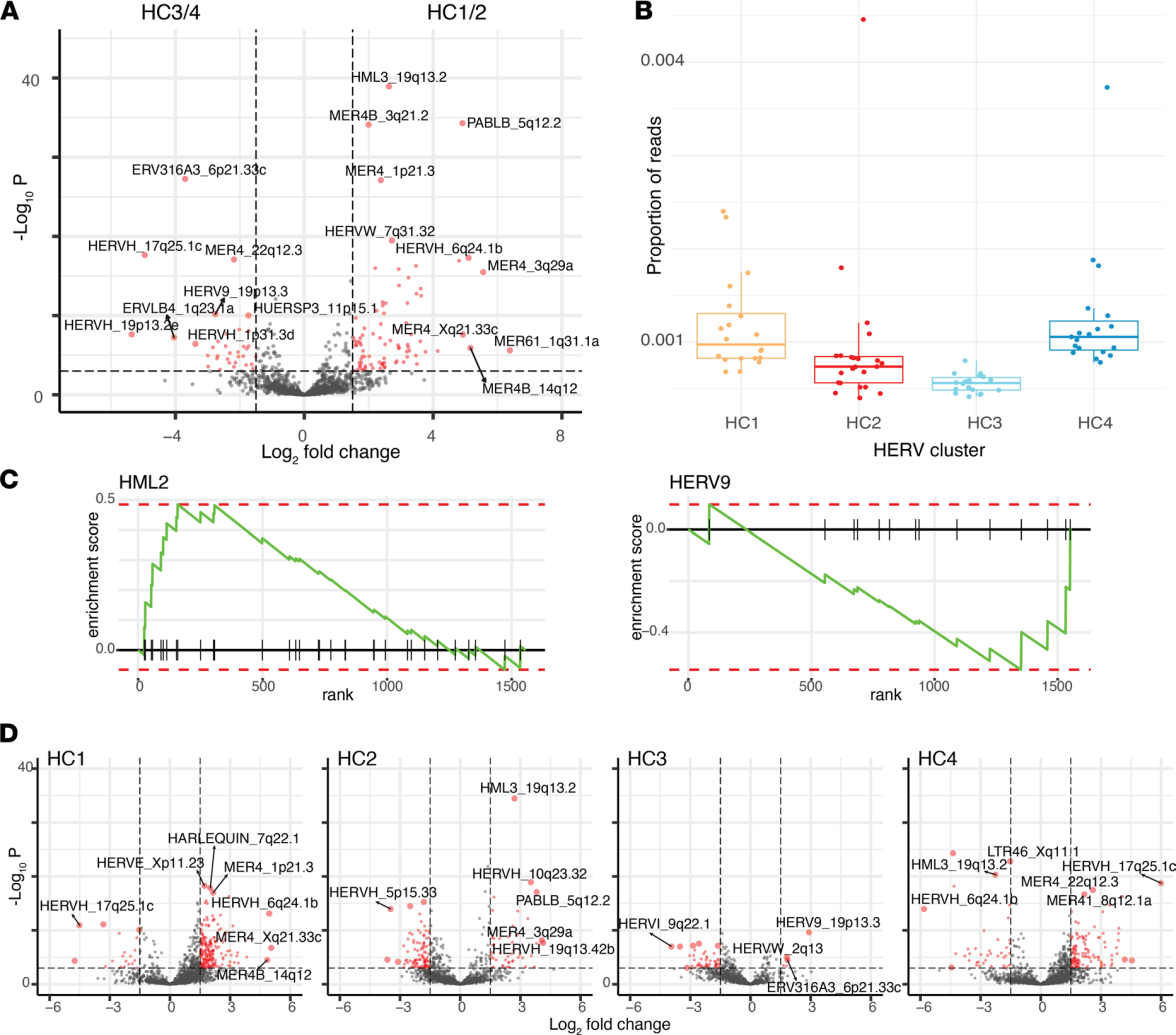
Differential HERV expression among UM subtypes. (**A**) Volcano plot showing significance (-log_10_ [adj. p value]) and effect size (log_2_ [fold change]) for differences in HERV expression between UM subtypes HC1/2 and HC3/4. Dashed lines are shown at significance and effect size thresholds (FDR < 1e-3, abs [log_2_ fold change] > 1.5) and HERVs that meet both thresholds are shown in red. HERVs with the greatest significance or effect size are labeled. (**B**) HERV transcriptional burden represented by overall proportion of reads that originate from HERV transcripts, by cluster. (**C**) GSEA enrichment plots for HML2 and HERV9 families. All genes are ordered by rank with vertical ticks indicating membership within the gene set; the running sum enrichment statistic is shown in green; and red dashed lines indicate the maximum and minimum enrichment scores. (**D**) Volcano plots, as in A, showing HERV significance and effect size significant HERVs for each cluster. Each plot shows the contrasts for 1 cluster compared with the average of the other 3 HERV clusters.

Having identified UM subsets based on the overall HERV expression landscape, we next asked which specific HERV loci were responsible for differences among subsets. First, we tested for HERVs that were differentially expressed between good prognosis (HC1/2) and poor prognosis subsets (HC3/4). We identified 86 HERVs that were significantly upregulated in HC1/2, while 34 HERVs were activated in HC3/4 (FDR < 1 × 10^–3^, abs [log_2_ fold change] > 1.5) (Figure 3A). The overall HERV burden, as measured by the proportion of all sequencing fragments originating from HERV loci, was greatest in HC4 (0.12%) and smallest in HC3 (0.055%) but was similar between HC1/2 (0.10%) and HC3/4 (0.091%) (Figure 3B). Gene set enrichment analysis (GSEA) revealed that HERVs overexpressed in HC1/2 were significantly enriched on chromosomes 3 and 19, while HERVs upregulated in HC3/4 were enriched on chromosome 8 (FDR < 0.01) (Supplemental Figure 1; supplemental material available online with this article; https://doi.org/10.1172/jci.insight.147172DS1). Family-level analysis revealed that significantly upregulated HERVs in HC1/2 were from 33 HERV families; several families were overrepresented including HERV-K families HML2, HML3, HML4, HML5, HML6, and HERVKC4. HC3/4 had upregulated loci from 21 families, and HML2 and HML5 were also overrepresented, as well as HERV9 and HERVI. GSEA with HERV family gene sets did not find any significantly enriched families (Supplemental Figure 2). Examination of HML2 and HERV9 enrichment plots revealed that gene set members were enriched on both ends of the ranking metric, indicating that individual loci are regulated independently as opposed to family-level regulation (Figure 3C).

Specific HERV loci were found to be characteristic of each subset compared with the other 3 subsets. HC1 had the greatest number of upregulated HERV loci (201 loci), followed by HC4 (118 loci), HC2 (47 loci), and HC3 (5 loci) (Figure 3D).

### HERV signature predicts UM subset.

**Figure 4:**
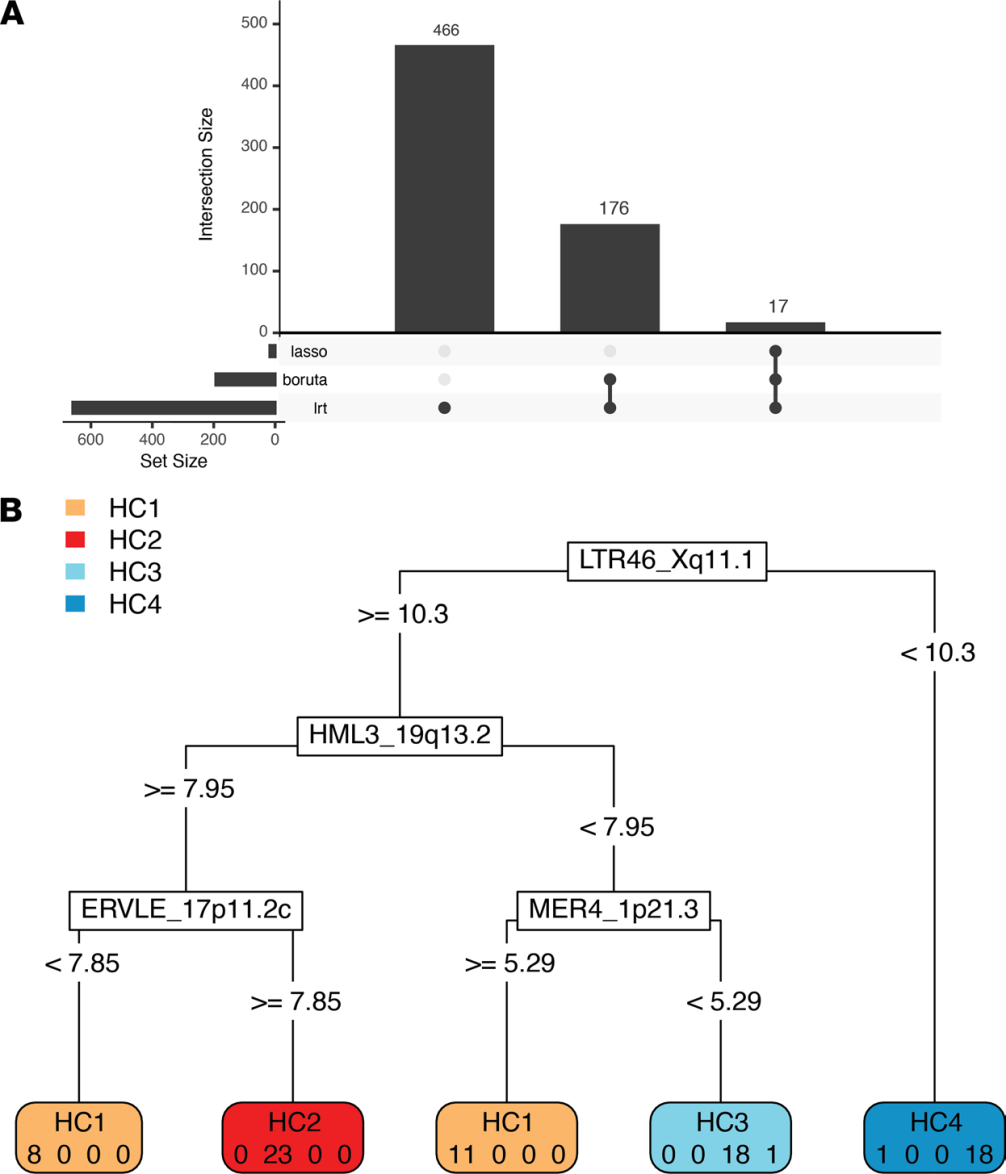
Prognostic HERV model using supervised learning. (**A**) Upset plot showing unique and shared features selected using the LRT, Boruta algorithm with random forest classification, and randomized LASSO with stability selection. Solid dots on the lower plot indicate algorithms involved in the intersection, and the height of the bar plot is the number of intersecting features selected. (**B**) Classification tree grown by recursive partitioning based on the 17-HERV signature identified by all algorithms. The HERV locus corresponding to each split is shown with normalized and transformed cutoff values shown on the branches. Final classification is shown on the leaf nodes with the “true” class assignments shown below. For example, samples classified into the leftmost node would be assigned to HC1. Of those samples classified to that node, 8 were actually from HC1 and 0 were from HC2, HC3, and HC4, respectively.

We asked whether we could identify a prognostic HERV signature that could be used to classify primary UM tumors. Our goal is to reduce the large number of HERVs deemed significant by differential expression testing, thereby narrowing targets for further investigation. We used 2 supervised learning approaches for evaluating the importance of HERV loci: random forest classification with the Boruta algorithm, which aims to identify all relevant features; and randomized least absolute shrinkage and selection operator (LASSO) regression, which selects a minimum optimal set of features. Differential expression testing using the likelihood ratio test (LRT) identified 659 loci with significant (FDR < 1 × 10^–3^) differences between a model that includes the HERV subtype compared with a null model. Machine learning approaches identified a much smaller set of important features, with 193 HERVs selected by Boruta and 17 using LASSO. Features selected with LASSO are a proper subset of Boruta-selected features and both approaches are subsets of significant LRT features (Figure 4A).

Using the features identified by each approach, we built multiclass support vector machine (SVM) classifiers to measure the accuracy of the HERV signature in predicting UM subtypes. SVMs were trained with repeated 5-fold cross-validation on the set of HERVs selected by each approach. All HERV signatures had a greater than 95% classification accuracy. The minimal optimal feature set (selected by LASSO) achieved 97.2% accuracy with a 17-HERV signature, while the Boruta and LRT signatures achieved 98.2% and 96.7% accuracy, respectively. In terms of per-class performance, HC1 was misclassified most frequently (recall = 0.919) while other classes were more sensitive (recall HC2 = 0.973; HC3 = 0.999; HC4 =0.9995). Misclassification among good prognosis and poor prognosis clusters was rare and almost exclusively due to misclassification of HC1 subtypes as HC4. We constructed a classification tree based on the 17-HERV signature that correctly classified 97.5% of cases (Figure 4B). HC4 cases were characterized by low expression of LTR46_Xq11.1, while HC3 had low expression of both HML3_19q13.2 and MER4_1p21.3. HC2 samples had high expression of ERVLE_17p11.2c.

**Figure 5:**
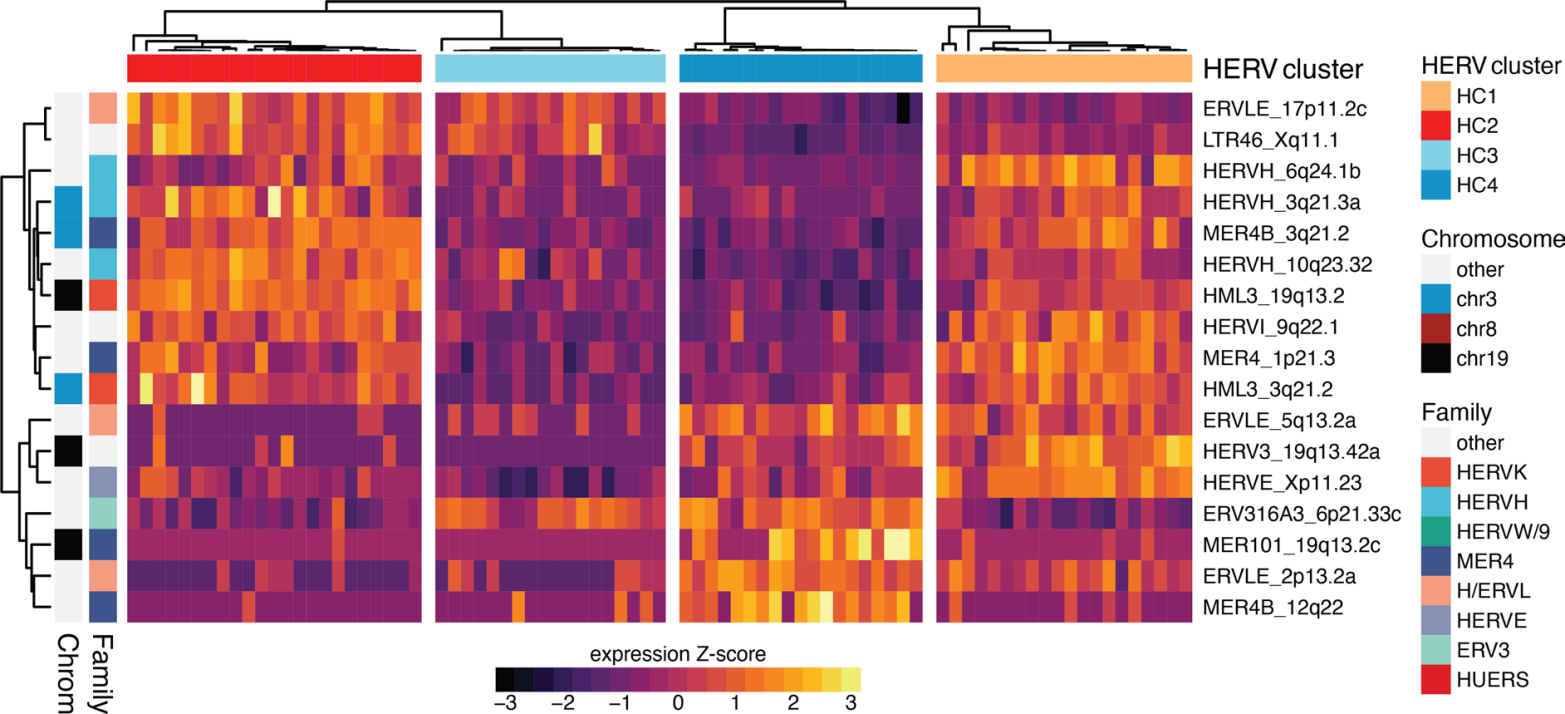
HERV expression signature distinguishes UM subsets. Heatmap showing HERV expression profiles for the minimal optimal set HERVs (17-HERV signature). Heatmap rows, representing HERV loci, are ordered by unweighted pair group method with arithmetic mean (UPGMA) hierarchical clustering of rank-based (Spearman’s) correlation distance and scaled to 0 mean and unit variance. Rows are annotated according to HERV locus categorization by chromosome and HERV superfamily. Columns are ordered by unsupervised consensus clustering of HERV profiles as previously described and annotated by HERV cluster assignment.

We have shown that our classification of UM based on HERV profiles is accurate and have identified a HERV signature with key marker HERVs for each UM subtype. However, the functional importance of specific HERV families or loci is largely undescribed. Thus, we sought to further characterize these HERVs and their potential products. HERVs selected for the 17-HERV signature were located in intergenic, exonic, and intronic regions and had various capacities for encoding proteins (Table 1). The 3 HERVs located in band 3q21 — HERVH_3q21.3a, MER4B_3q21.2, and HML3_3q21.2 — are downregulated in HC3/4 UMs (Figure 5). This subtype is associated with the class 2 high metastatic risk UMs that have recurrent loss of 1 copy of chromosome 3 (M3). None of the HERVs used in our classifier were located on chromosome 8, despite the association of increased chromosome 8q copy number with HC4. Of those HERVs included in the signature, 7 loci have significant (FDR < 1 × 10^–3^) expression differences distinguishing poor prognosis cluster HC3 from HC4. HERVH_6q24.1b, HERVH_10q23.32, ERVLE_17p11.2c, and LTR46_Xq11.1 have higher expression in HC3, while ERVLE_2p13.2a, ERVLE_5q13.2a, and HERVE_Xp11.23 are more expressed in HC4.

### Predicted HERV-derived T cell antigens.

Cytotoxic T cells recognize peptide fragments of proteins presented in the groove of the MHC molecule, and which peptides are selected depends upon donor haplotype. Class I restricted peptides are approximately 9 amino acids in length, while class II are 11–14 amino acids. In cutaneous melanoma, certain peptides have been targeted for cytotoxic T lymphocyte (CTL) expansions, and HERV-derived peptides have been identified as infectious disease targets. From our analysis, we then determined the potential T cell epitope region, concentrating on proteins from open reading frames (ORFs) in the 17-HERV signature we found differentially upregulated from the Telescope analysis. We identified 3 HERV loci with protein-coding potential based on intrinsic sequence composition: MER4_1p21.3, HML3_19q13.2, and HERVE_Xp11.23. We identified ORFs and characterized the resulting sequences by sequence similarity with other retroviral proteins (Supplemental Table 1). MER4_1p21.3 contained 2 ORFs that did not share similarity with retroviral proteins, while HML3_19q13.2 and HERVE_Xp11.23 contained partial coding sequences for retroviral genes *gag*, *pol*, and *env*.

To map epitopes to short peptides we used netMHCpan (68) to look for peptides from ORFs for common HLA alleles in the TCGA-Uveal Melanoma (TCGA-UVM) cohort. For each of the HLA alleles, amino acid sequences were scanned for peptides of length 9 and were scored according to the likelihood of the peptide being an MHC ligand. Peptides with percentile rank_EL less than 0.5 were considered to be “strong binders.” We report the number of strongly binding peptides for each HLA allele and ORF combination and the amino acid sequences for all strong binders (Supplemental Tables 2 and 3). Across all alleles, 143 strongly binding peptides were identified for HERVE_Xp11.23, 85 for HML3_19q13.2, and 9 for MER4_1p21.3.

### HERV subtype classifier in UM cell lines.

We evaluated the applicability of our classifier using publicly available UM RNA-Seq data sets. We identified 65 samples from several studies (69–73) (Supplemental Table 4; GEO Series GSE176345, NCBI BioProject PRJNA59636) and used Telescope to profile HERV expression. Fragment counts were normalized for library size together with TCGA samples using DESeq2. Initial analysis of the combined data set with principal component analysis (PCA) (2872 features) reveals high variability distinguishing TCGA from other data sets (Supplemental Figure 4). Since these data sets examined different tumors or cell lines and were obtained using different protocols, we cannot rule out technical or biological explanations for these differences. To overcome these pitfalls, we relied only on the 17 HERV loci determined to be most informative for subtype classification. Assignment of cell line samples to HERV subtype clusters was less confident than in TCGA cases; the greatest assignment probability in cell lines was 48.2%, while TCGA probabilities ranged from 60.7% to 97.6%. Out of 30 samples belonging to the Mel202 cell line, 26 were classified in the HC4 cluster. Similarly, all 4 Mel270 samples were classified as HC4, while samples from 92.1 were assigned to HC1. Out of 65 samples, all except 13 were assigned to HC4. We examined expression at LTR46_Xq11.1 more closely since this feature has the greatest variable importance in our model. Average expression at this locus was much lower in other studies compared with TCGA; low expression values result in HC4 classification. Visual examination of other HERV loci revealed a similar pattern for some loci, while others had average expression more comparable to TCGA (Supplemental Figure 5).

### BAP1 mutant UM activates specific HERV expression programs.

BAP1 is a deubiquitinating enzyme with an increasingly recognized role in tumor suppression. In UM, metastatic risk groups are be stratified by BAP1 status, with class 2 “poor prognosis” UMs associated with loss of BAP1. We sought to investigate the association between inactivating mutations in BAP1 with HERV expression by identifying HERV loci that are differentially expressed in BAP1 WT (BAP1wt) and mutant (BAP1mut) tumors (Supplemental Figure 6). In the TCGA cohort, BAP1mut UMs (*n* = 35) had 22 HERV loci that were activated and 67 that were downregulated compared with BAP1wt UMs (*n* = 45). In UM cell line expression data from Han et al. (71) (GEO accession GSE149920), BAP1wt cell lines (MM66) had 33 upregulated and 29 downregulated HERVs compared with BAP1mut cell lines (MP38, MP46, MP65 and PDX4). Five loci — HERVH_1p31.3d, ERV316A3_6p21.33c, MER4_22q12.3, MER4B_8q21.11, and HERVH_5p15.33 — were upregulated in BAP1mut UMs for both cohorts. Of these, ERV316A3_6p21.33c was also important as a feature in the 17-HERV classification signature.

## Discussion

In this study, we have characterized the HERV expression landscape in primary UM tumors and show that HERV expression profiles are useful for distinguishing metastatic outcomes in patients with UM. Unsupervised learning shows that HERV profiles define 4 UM subsets that provide clear delineation of risk outcome and support subtypes identified by other molecular indicators. The concordance of HERV-based UM subtypes with other “-omics” classifiers suggests that distinct UM subtypes are driven by systems-level perturbations manifested through multiple indicators. Not surprisingly, this correlation was strongest with other transcriptome-based platforms, including mRNA and lncRNA profiles. However, as the vast majority of HERV loci identified in our annotation are excluded from widely used genome annotations, retrotranscriptomic studies have the potential to identify novel RNA species that would be overlooked using established transcriptomic approaches.

Identifying differential expression of HERVs among varying prognostic subtypes has 2 broad implications. First, it suggests that distinct HERVs are associated with the risk of UM metastasis and may offer mechanistic insights into this process. This is supported by the finding that the better-prognosis HC1/2 clusters were enriched with upregulated HERVs on chromosome 3 and poor-prognosis HC3/4 clusters were enriched with upregulated HERVs on chromosome 8, consistent with the poor prognosis of M3 and 8q amplification. The 3 HERV elements in 3q21 were of particular interest as their expression patterns were highly informative for classification. It remains to be seen whether this aberrant expression is directly related to M3 or related to global methylation changes resulting from loss of BAP1. Enrichment of DE HERVs on chromosome 19 in HC1/2 additionally suggests that dysregulation of HERVs on chromosome 19 may be associated with metastatic potential. Second, HERV loci characteristic of high-risk UM subtypes could represent underappreciated targets for immune-based therapy.

Our HERV-based UM classifier is a proof-of-concept that patterns of HERV expression may be used to develop biomarkers useful for UM classification and prediction of clinical outcomes. The minimum optimal feature set was able to achieve greater than 95% accuracy for 4 subset classifications using only 17 HERVs. This signature was highly sensitive, with greater than 99% of poor prognosis HC3/4 UMs correctly classified as such. We constructed a classification tree from this 17-HERV signature that uses only 4 HERV loci and that correctly classifies 97.5% of cases. At the very least, these classifiers provide useful suggestions for HERV loci, warranting further investigation as potential biomarkers or neoantigens. We tested our classifier on out-of-sample data from UM cell lines and found that most samples were assigned to HC4. The locus with the highest variable importance in our model, LTR46_Xq11.1, appeared to have much lower expression in cell line data (Supplemental Figure 5), resulting in low-confidence assignments to HC4. Given that cell lines do not necessarily maintain the same expression patterns as the primary cells from which they are derived, biological differences affecting HERV expression are plausible, as are technical sources of variation. Ideally, out-of-sample data from primary UM cases, generated using standardized protocols, will become available for testing our model.

Specific HERV upregulation has previously been associated with responsiveness to immune checkpoint blockade in patients with renal cell cancer (29, 55), and a novel HERV-E neoantigen was identified (54). The restricted expression of HERV-E in kidney tumors was found to occur as a consequence of inactivation of the von Hippel–Lindau tumor suppressor. Antigens derived from this provirus are immunogenic, stimulating cytotoxic T cells that kill kidney cancer cells in vitro and in vivo. In this study, we identified an intergenic HERV on the X chromosome (HERVE_Xp11.23) that potentially encodes immunogenic peptides and is highly expressed in HC4 poor-prognosis UMs, a possible immunotherapeutic target.

The tumor microenvironment and immune landscape of primary UM has been found to be an important determinant of UM subtype and prognostic outcome. In contrast to many other cancers, immune infiltration in UMs tends to be associated with worse prognosis (74). Our study suggests that increased infiltration (Supplemental Figure 3) is also associated with higher overall HERV burden in HC4 UMs and increased expression of specific HERV loci, including HERVE_Xp11.23. Our MHC class I binding analysis revealed numerous ORFs encoding epitopes that may be bound by several HLA alleles. Since tebentafusp can only be used in individuals with the HLA-A2 allele (40–50% of patients), identification of potential epitopes restricted by other HLA alleles could broaden antigen selection for future immunotherapeutic approaches for all patients. Additional studies examining the complex relationship between immunogenic HERV burden, immune infiltration, and prognostic outcome are important to advance the field.

There are some limitations to our study, including the scarcity of available data sets and no RNA-Seq data from UM liver metastases. Identification of which HERVs are DE in liver metastases compared with the primary tumor may allow rational antigenic targeting.

In summary, our work demonstrates that HERV expression quantified at the family level may prove to be overly simplistic to accurately describe HERV activity, while locus-specific HERV profiling can better account for independent expression of HERV insertions due to sequence variation, local genomic context, or epigenetics. Together, these studies suggest that defining locus specific HERV expression in uveal melanoma may provide insights into oncogenesis and metastases, response to treatment, and creation of potentially novel avenues of therapy.

## Methods

### Data processing.

The NCI’s Genomic Data Commons (GDC) (75) was used to identify 80 primary UM tumors with available RNA-Seq data from the TCGA-UVM project. Paired-end RNA-Seq data (Illumina) were downloaded in Binary Alignment Map (BAM) format using the GDC data transfer tool. Aligned BAM files were reverted to unaligned uBAM files and adapter sequences were marked using Picard (76). Adapter-trimmed reads were aligned to the reference genome hg38 (GCA_000001405.15 no-alt analysis set) using bowtie2 (77) with very sensitive local alignment, reporting up to 100 alignments per read with approximately 95% or greater sequence identity (--very-sensitive-local -k 100–score-min L,0,1.6). The resulting BAM files containing ambiguous reads were reassigned using Telescope (67) with the “retro.hg38.v1” annotation obtained from (https://github.com/mlbendall/telescope_annotation_db/tree/af3c359/builds/retro.hg38.v1). This annotation includes 14,968 HERV and 13,545 L1 loci. Telescope was run with up to 200 iterations of expectation-maximization and an informative prior for theta (--max_iter 200–theta_prior 200000). The final read counts output by Telescope were used in downstream retrotranscriptome analysis.

Gene expression was estimated using a pseudoalignment approach. The transcriptome index was built using the GENCODE v22 annotation obtained from GDC and adapter-trimmed reads were input to kallisto (78) with 100 bootstrap samples (-b 100).

The data processing pipeline was implemented on a high-performance computing cluster using Snakemake (79) with the bioconda package manager (80). The complete analysis pipeline is available from (https://github.com/mlbendall/herv_melanoma).

### Patient characteristics, clinical outcomes, and molecular characterization.

Patient characteristics and clinical outcomes for 80 UM samples were obtained from NCI GDC (81) and TCGAbiolinks (82). Additional clinical data and molecular characterization, such as cluster assignments and somatic mutations, were previously reported and obtained from Robertson et al. (8). Leukocyte fraction and CIBERSORT immune fractions were estimated by Thorsson et al. (83) and accessed through the NCI’s GDC.

### Unsupervised analysis.

Unsupervised analysis was performed using the R statistical environment and Bioconductor package manager (84). HERV counts reported by Telescope were filtered to exclude any loci that had fewer than 5 observations (fragments) across all samples, resulting in profiles of 4104 HERV loci. Counts were normalized using size factors calculated by DESeq2 (85) and transformed using a variance stabilizing transformation. PCA was performed on the transformed counts and visualized using PCAtools (86). Consensus clustering with the k-means clustering algorithm was performed for 1000 replicates using ConsensusClusterPlus (87). The clustering procedure was performed for k = 2 through k = 9; the resulting cluster solutions were evaluated using consensus matrices and silhouette statistics. Clustering solutions for k = 2, k = 3, and k = 4 appeared to describe the data well and yielded clusters of sufficient size, and a final clustering of k = 4 was chosen based on agreement with other molecular and clinical indicators. Dendrogram was created with the dendextend package (88). Kaplan-Meier survival curves were fitted using survival (89) and drawn using survminer (90). Intervals to clinical outcomes were calculated for documented death due to mUM or last follow-up and were censored at 5 years. The adjusted Rand index was calculated to compare clustering solutions and Fisher’s exact test was used to test for significant correlations between HERV clustering and categorical variables.

### Differential expression analysis.

Differential expression analysis was performed using the R statistical environment using the DESeq2 package (85). The experimental model included 1 term for HERV cluster assignment with 4 levels (~ clust.herv + 0). Comparison between poor and good prognosis clusters were made by comparing the average of C1 and C2 to the average of C3 and C4, i.e., ([C1+C2)/2] — [C3+C4)/2]). Cluster-specific contrasts were extracted for each HERV cluster compared with the mean of the other 3 clusters, i.e., C1 — ([C2+C3+C4]/3). Thresholds for significant genes were FDR less than 1 × 10^–3^ and abs (log2 fold change) greater than 1.5. Significance and effect size were visualized using volcano plots implemented in the EnhancedVolcano package (91). Heatmap and upset plot visualizations were implemented in pheatmap (92) and UpSetR (93), respectively.

Gene sets for GSEA were created using the retro.hg38.v1 annotation. HERV loci were grouped into families according to the internal region. The test statistic calculated in DESeq2 was used to rank genes. GSEA was performed using the adaptive multilevel splitting Monte Carlo approach implemented in fgsea (94).

### Supervised learning and prognostic HERV model.

Supervised analysis was performed using the R statistical environment. HERVs used for the LRT and supervised learning were filtered to exclude loci that were not observed (fragment count threshold greater than 5) in at least 5% of samples, resulting in 1122 loci. The LRT was used to compare the experimental design including HERV cluster (~ clust.herv) to a reduced model (~ 1) with a significance threshold of FDR less than 1 × 10^–3^. Fragment counts were normalized and transformed (variance stabilizing transformation) with DESeq2 and the transformed matrix was used for feature selection. Minimum optimal feature selection was performed using randomized LASSO regression with stability selection, described by Meinshausen and Bühlmann (95) and implemented in glmnet (96, 97) and c060 (98). LASSO was fit using multinomial logistic regression with grouped penalty that ensures multinomial coefficients for a variable are either all non-0 or all 0 (a feature is either all-in or all-out). Randomization was performed by reweighting features in each subsample by a random weight in (0.1, 1) (weakness = 0.1), and 100 subsamples were used for stability selection. Stable features were selected with a proportion threshold of 0.6 and an error rate of 0.05 (error = 0.05, pi_thr = 0.6). All relevant feature selection was performed using the Boruta algorithm (99) with random forest classifiers from the randomForest package (100). Random forests were grown with 1000 trees (ntree = 1000) and 1000 importance runs were performed (maxRuns = 1000). A small number of orutave variables after 1000 iterations were fixed by comparing the median importance with the median importance of the maximal shadow attribute. UpSetR was used to visualize overlap of features selected using the 3 approaches.

HERV signatures were tested by constructing support vector machines, implemented in e1071 (101) using the selected feature set with 5-fold cross-validation and 100 repetitions. Accuracy was calculated for each fold and each repetition and mean accuracy was taken as the average across all folds and repetitions. A classification tree was grown by recursive partitioning (102) based on the LASSO signature (17 variables).

### Locus-specific HERV characterization.

Meta-annotations of locus-specific HERVs were obtained from https://github.com/Liniguez/Telescope_MetaAnnotations Protein-coding potential of HERV elements was calculated based on intrinsic sequence composition using the Coding-Non-Coding Identifying Tool (CNIT) webserver (103). The nearest gene or gene overlaps were determined using the ENSEMBL HG38 annotation, release 99.

### HERV immunogenicity prediction.

ORFs were identified using Geneious with minimum size of 200 nt and start codons ATG; only ORFs in the sense orientation relative to the HERV locus were retained. Possible peptides with homology to retroviral proteins were identified using a protein-protein blast (blastp) search against NCBI nonredundant protein sequences (nr) database, limited to records assigned by Retroviridae (taxid: 11632). All ORFs were used to predict peptides with high binding affinity to MHC class I molecules using NetMHCpan version 4.1b. We tested binding affinity to HLA class I alleles with 10 or more samples in the TCGA-UVM cohort as predicted by Thorsson et al. (83). HLA-A alleles include HLA-A*02:01, HLA-A*03:01, HLA-A*01:01, HLA-A*11:01, and HLA-A*24:02. HLA-B alleles include HLA-B*44:02, HLA-B*07:02, HLA-B*18:01, and HLA-B*08:01. ORFs were analyzed using a peptide length of 9. Strong binders were selected using the percentile rank of the elution ligand score (%Rank_EL < 0.500).

### Statistics.

Differential expression significance was calculated using the Wald test, and multiple test correction was performed using the Benjamini-Hochberg procedure. Feature selection was performed using 3 methods: likelihood ratio test, Boruta random forest algorithm (99), and randomized least absolute shrinkage and selection operator (LASSO) regression. Multiclass support vector machine (SVM) classifiers were trained using 5-fold cross validation and 100 repetitions.

## Author contributions

MLB performed the experiments. MLB, JHF, ANS, and DFN conceived the research. All authors wrote and approved the manuscript.

## Supplementary Material

Supplemental data

Supplemental table 1

Supplemental table 2

Supplemental table 3

Supplemental table 4

## Figures and Tables

**Table 1:**
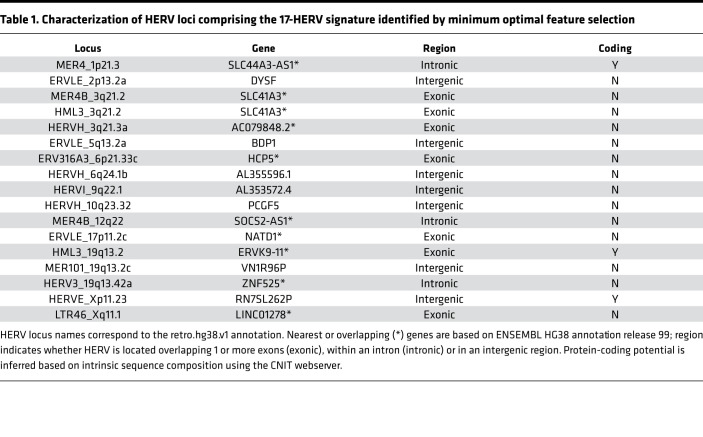
Characterization of HERV loci comprising the 17-HERV signature identified by minimum optimal feature selection

## Version 1 03/29/2022

In-Press Preview

## Version 2 05/09/2022

Electronic publication
