# Supplementary material for: Specific human endogenous retroviruses predict metastatic potential in uveal melanoma
Source: JCI Insight. 2022 May 9;7(9):e147172. doi: 10.1172/jci.insight.147172 (PMC9090245; doi:10.1172/jci.insight.147172)
Supplement: Supplemental data [file jciinsight-7-147172-s109.pdf]

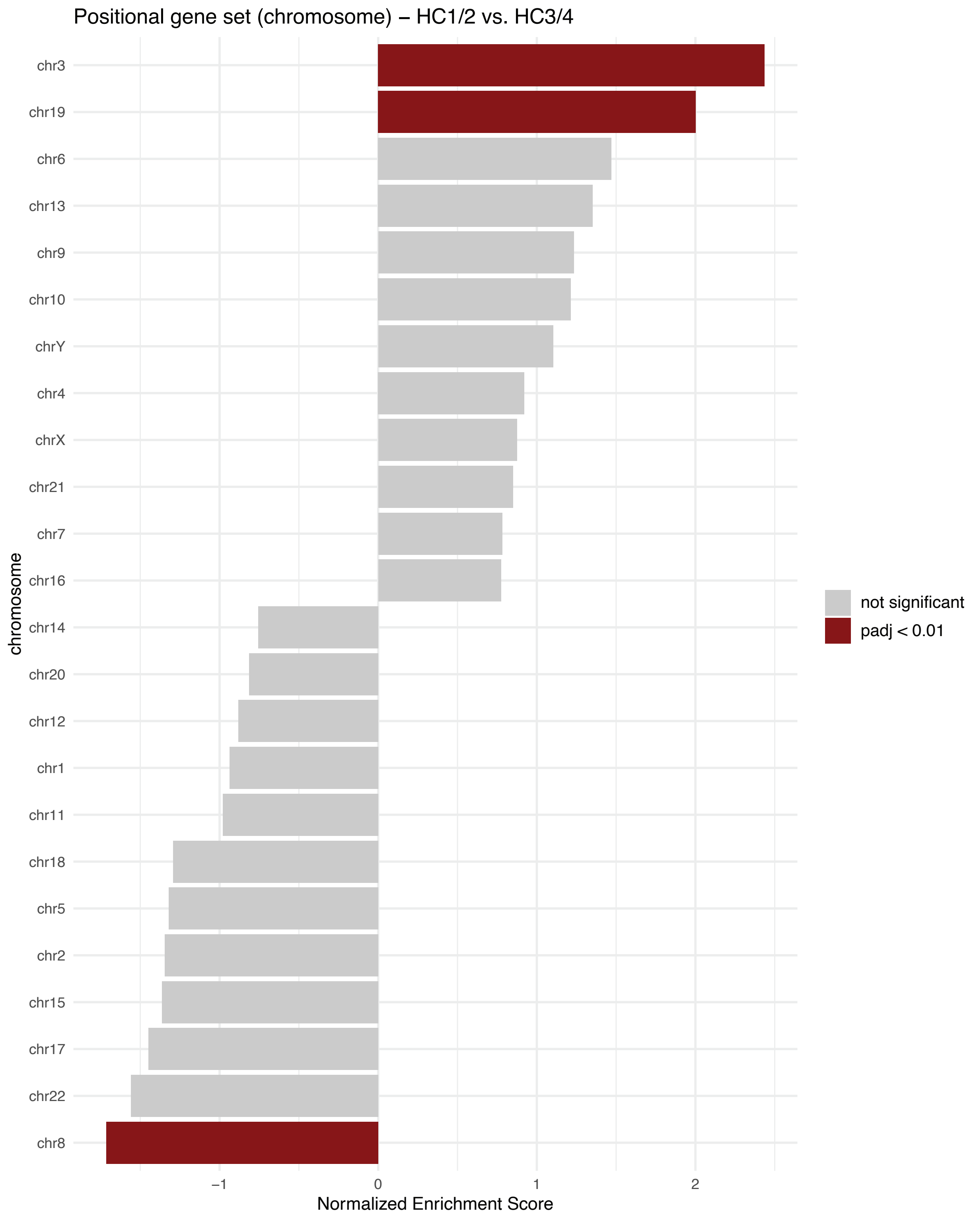

Supplemental Figure 1. Relative enrichment of HERV expression in UM subtypes based on positional gene sets. Gene set enrichment analysis was performed using positional gene sets where each HERV locus is a member of the chromosome set on which it is located. Chromosomes that are enriched in HC3/4 have positive normalized enrichment scores (NES > 0), while negative enrichment scores indicate chromosomes that are enriched in HC1/2. The test statistic calculated in DESeq2 was used to rank genes. GSEA was performed using the adaptive multilevel splitting Monte Carlo approach implemented in fgsea(94).

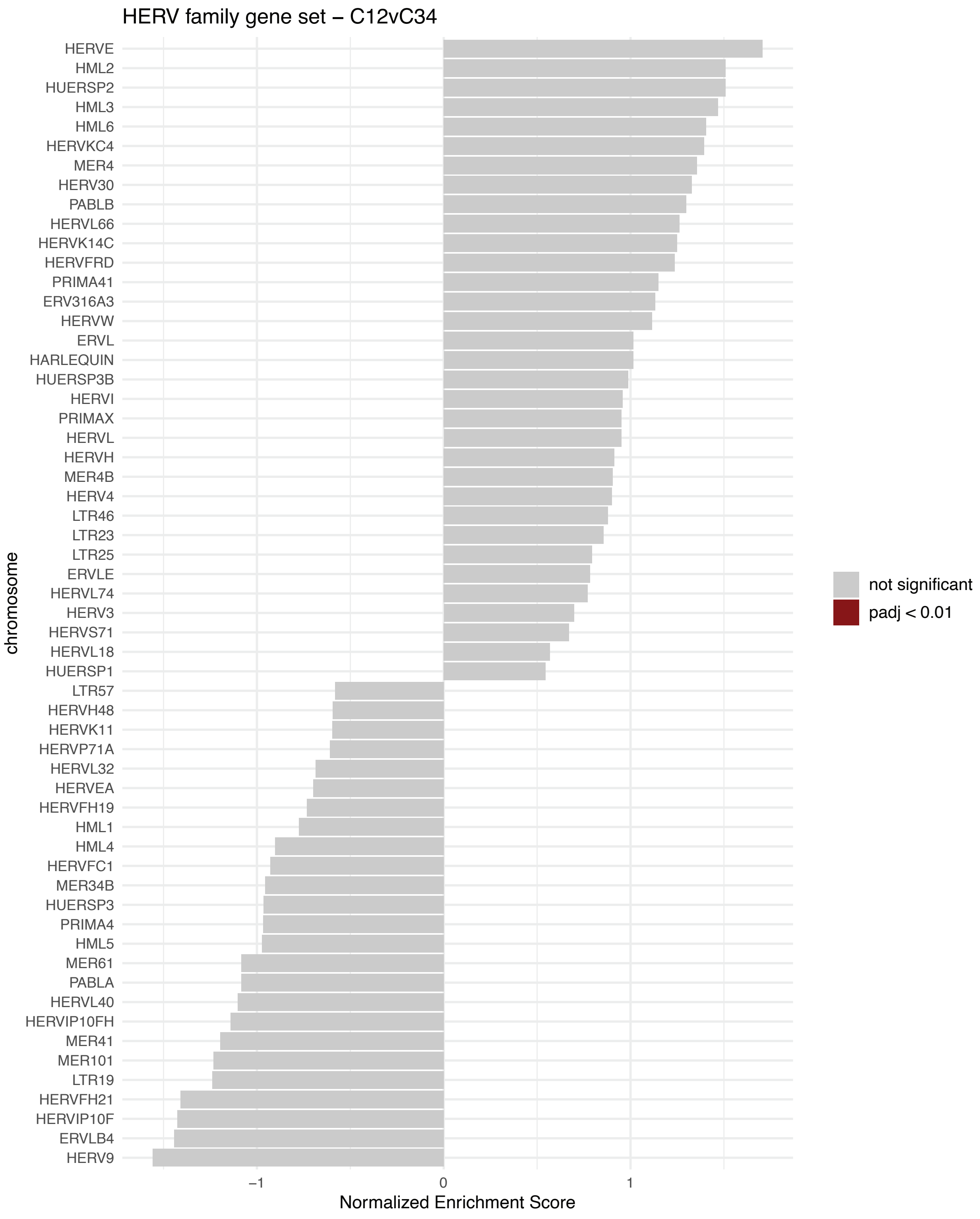

Supplemental Figure 2. Relative enrichment of HERV expression in UM subtypes based on HERV family. Gene set enrichment analysis was performed using HERV family gene sets where each HERV locus is grouped into gene sets based on the family identity of the internal region. Chromosomes that are enriched in HC3/4 have positive normalized enrichment scores (NES > 0), while negative enrichment scores indicate chromosomes that are enriched in HC1/2. The test statistic calculated in DESeq2 was used to rank genes. GSEA was performed using the adaptive multilevel splitting Monte Carlo approach implemented in fgsea(94).

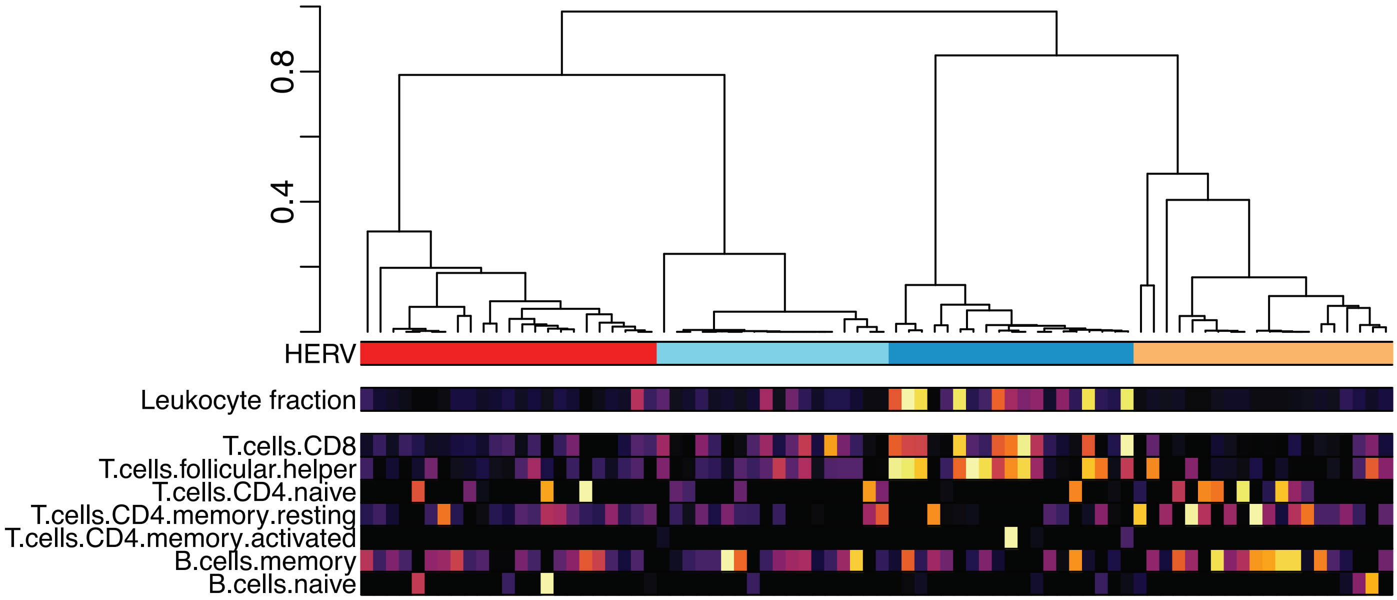

Supplemental Figure 3. Immune landscape of HERV-based clusters. Clustering dendrogram of HERV profiles with covariate tracks indicating (from top) leukocyte fraction, CD8+ T cells, T follicular helper cells, naive CD4+ T cells, resting memory CD4+ T cells, activated memory CD4+ T cells, memory B cells, and naive B cells. Leukocyte fraction and CIBERSORT immune fractions were estimated by Thorsson et al.(83) and accessed through the NCI Genomic Data Commons.

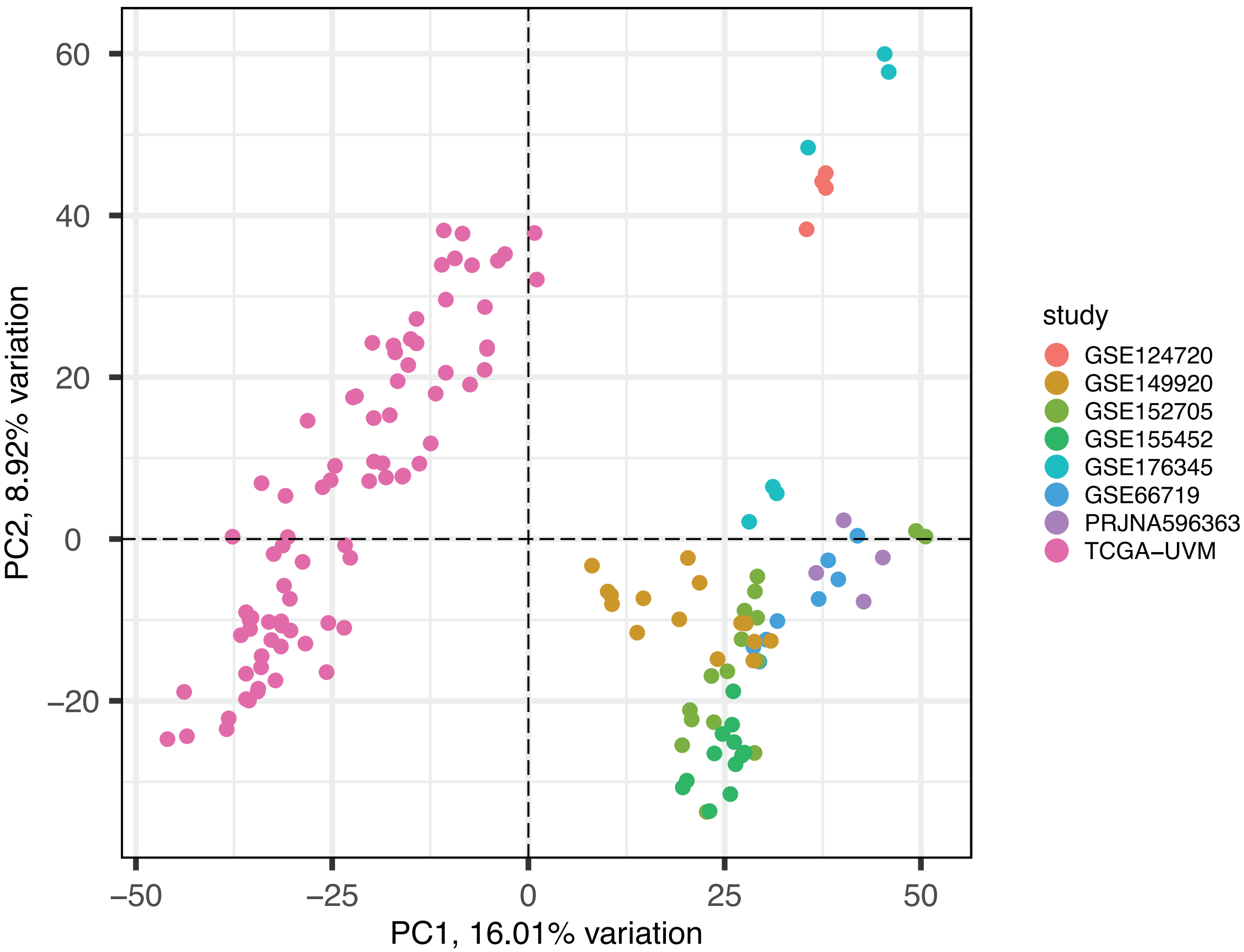

Supplemental Figure 4. Principal component analysis of HERV profiles from TCGA and cell line datasets. Raw read counts for all samples were normalized together using DESeq2(85). Principal component analysis was performed using PCATools(86).

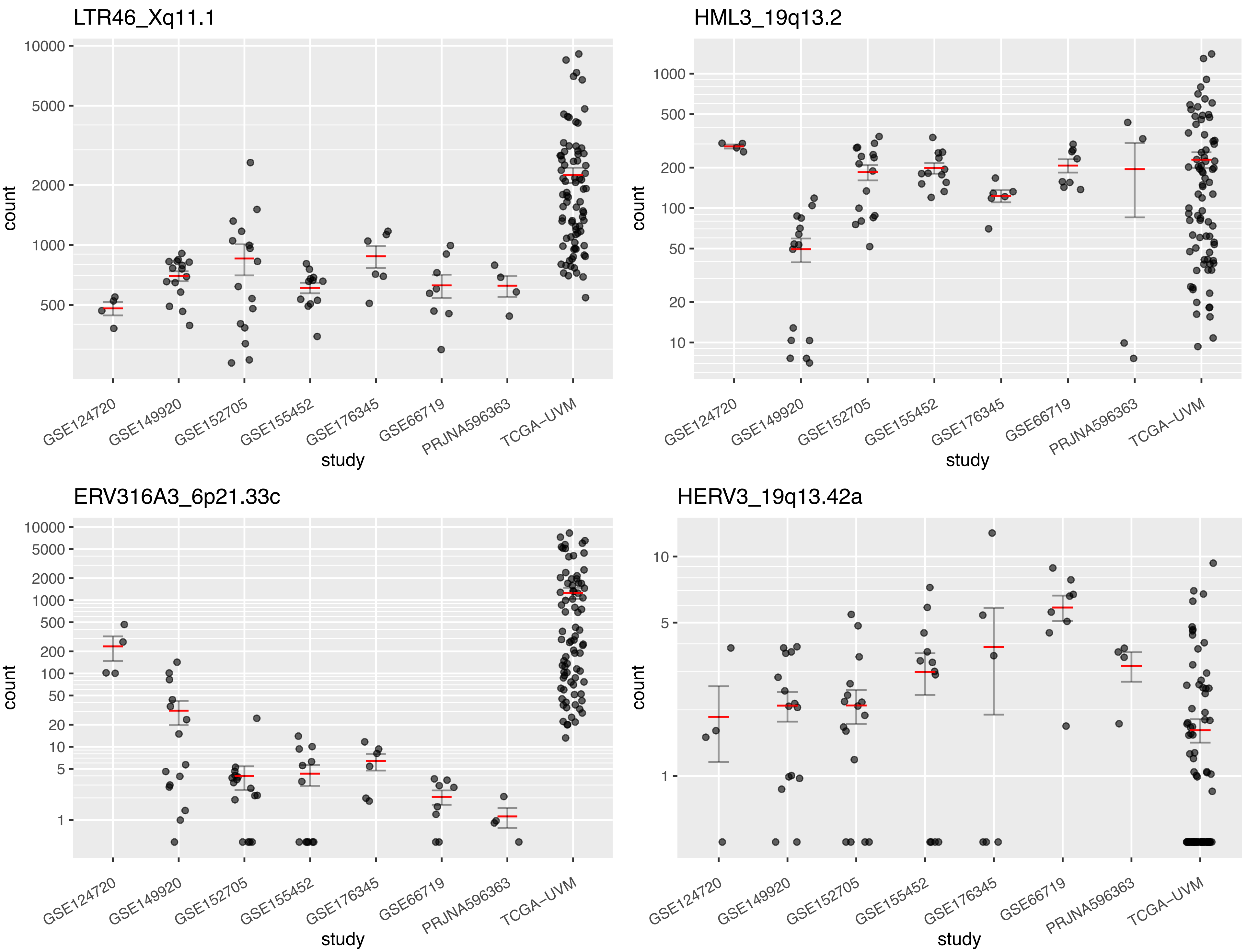

Supplemental Figure 5. Normalized counts in HERVs with high variable importance. Normalized counts are shown for four representative loci: LTR46_Xq11.1, HML3_19q13.2, ERV316A3_6p21.33c, and HERV3_19q13.42a. The mean and standard error within each study is shown.

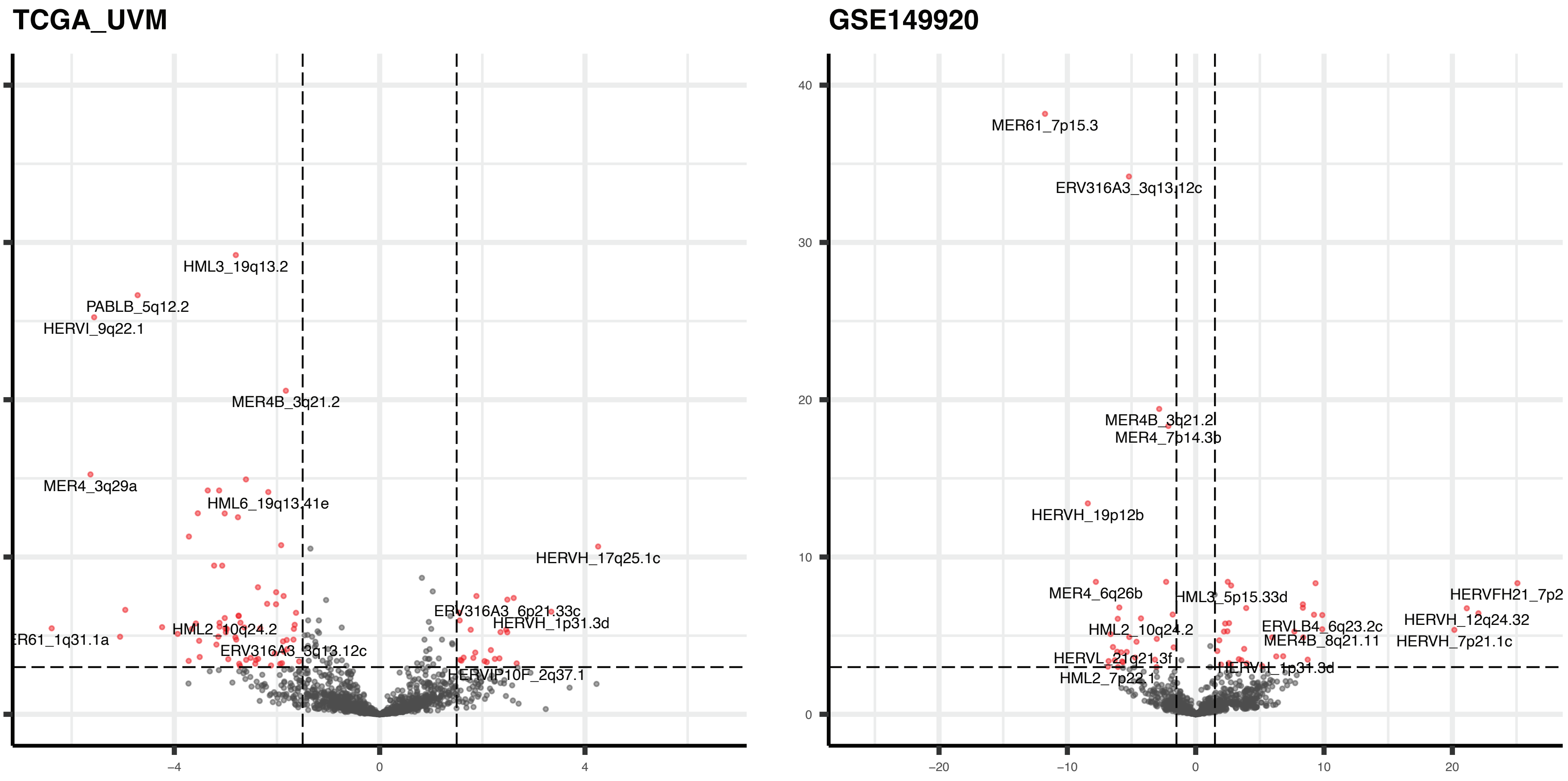

Supplemental Figure 6. Volcano plot of differentially expressed HERVs in BAP1 wild-type compared to BAP1 mutant UM. Positive log2FoldChange values (x-axis) indicate upregulation in BAP1mut relative to BAP1wt. TCGA cohort compares 35 BAP1mut with 45 BAP1wt UM cases. GSE149920 compares MM66 BAP1wt cell line with BAP1mut cell lines MP38, MP46, MP65 and PDX4, three replicates each.
